# Potential role of Lu/BCAM in HIV-related atherosclerosis

**DOI:** 10.4102/ajlm.v8i1.792

**Published:** 2019-09-30

**Authors:** Modisa S. Motswaledi, Ishmael Kasvosve, Oluwafemi O. Oguntibeju

**Affiliations:** 1 Department of Biomedical Sciences, Faculty of Health and Wellness Sciences, Cape Peninsula University of Technology, Cape Town, South Africa; 2 Department of Medical Laboratory Sciences, Faculty of Health Sciences, University of Botswana, Gaborone, Botswana

**Keywords:** cholesterol, Non-high-density lipoprotein cholesterol, Lu/BCAM, Atherosclerosis

## Abstract

Atheromatous lesions are formed by macrophages and low-density lipoprotein cholesterol invading the vascular intima. Here we show that increasing cholesterol levels are associated with peripheral monocyte depletion and this imbalance is aggravated by carriage of Lu/BCAM leukocyte adhesion molecules. This is true only in HIV infection and probably explains the risk of atherosclerosis observed in HIV-positive patients.

## Introduction

The pathology of atherosclerosis is tightly linked to three main factors; leukocytes, plasma lipids and vascular injury or chronic inflammation.^1,2^ Leukocytes such as neutrophils, activated monocytes or monocyte-derived macrophages and platelets accumulate in the vascular sub-endothelial matrix by use of adhesion molecules. The Lutheran blood group, also known as the basal cell adhesion molecule (Lu/BCAM), is one such molecule and is expressed on both monocytes and endothelial cells.^3^ It is the natural ligand for laminin, an extracellular basement membrane protein that facilitates both adhesion and extravasation of monocytes.^3^ In addition, monocytes and neutrophils also constitutively express α4β1 integrin,^4^ another ligand for Lu/BCAM. These molecules enable these cells to adhere to the endothelium and have been implicated in the pathology of crescentic sickle cell disease,^4^ as well as in several cancers, suggesting more efficient tumour tethering and greater tumour size.^5,6^

In HIV-positive individuals, the viral Tat protein accumulates in the extracellular matrix of blood vessels and facilitates integrin-mediated adhesion^7^ with the consequence that HIV-infected monocytes have enhanced extravasation.^8^ HIV infection is also characterised by chronic inflammation resulting from the activation of T-cells, macrophages, neutrophils and natural killer cells – all of which release pro-inflammatory cytokines that create a state of persistent inflammation.^9^ This chronic inflammation involves neutrophils which produce reactive oxygen species thought to contribute to the oxidation of low-density lipoprotein cholesterol.^10^ Cholesterol enrichment of neutrophil membranes results in more stabilised rolling and adhesion on endothelial cells,^2^ where they may recruit monocytes^10^ to promote atherogenesis. In particular, non-high-density lipoprotein (HDL) cholesterol has been documented as one of the more important promoters of atherosclerosis in HIV-positive patients.^11^ The atheromatous lesion forms by gradual accumulation of oxidized low-density lipoprotein cholesterol and infiltration by macrophages, smooth muscle cells and platelets.^12^ This may ultimately narrow the vascular lumen, causing tissue hypoxia distal to the atheroma. Moreover, the release of RANTES (CCL5) by activated platelets in the atheroma leads to further recruitment of monocytes and neutrophils.^13^ A high monocyte count has thus been reported to be a long-term predictor of plaque formation,^14^ while neutrophil enzymes such as elastase and myeloperoxidase in the atheroma may destabilise it,^15^ leading to its fragmentation and subsequent generation of emboli that may cause vascular occlusion at distant sites.

In our previous study, the Lutheran blood group, Lu^b^, was associated with a three-fold risk for HIV infection,^16^ and it was suspected that this could be due to its adhesiveness, which promotes trans-endothelial migration and the spread of infected monocytes to distant sites. The current study therefore sought to determine the effect, if any, of carriage of the Lu^b^ antigen as an adhesion molecule on circulating monocytes and neutrophils, as well as how these parameters related to cholesterol measurements. The results indicate that HIV does affect the relationship between monocytes and cholesterol, and that this relationship is accentuated by expression of the Lu^b^ antigen.

## Methods

### Ethical considerations

Ethical clearance for the study was obtained from the University of Botswana’s Office of Research and Development (Permit No. URB/IRB/1365), Botswana’s Ministry of Health and Wellness Health Research Development Committee (Permit No. HPDME 13/18/1) and the Cape Peninsula University of Technology, Faculty of Health and Wellness Research Ethics Committee (Permit No. CPUT/HW-REC 2015/H11). Informed consent was not necessary, since de-identified, residual samples were used,^17^ and the study was approved to use residual samples.

### Study design

The study was conducted at the Julia Molefe Clinic, Gaborone, Botswana, from December 2016 to February 2017. One hundred blood samples comprising 58 female patients and 42 male patients were enrolled in the study. The patients had all tested positive for HIV and were being prepared for enrolment into the antiretroviral therapy programme. Patients were enrolled sequentially upon receipt of an adequate residual sample to perform requisite tests. A full blood count was done using the Sysmex^®^ XT1800i haematology analyser (Sysmex Corporation, Kobe, Japan). The samples were phenotyped for the Lutheran antigens (Lu^a^ and Lu^b^) using specific antisera (Fortress Diagnostics, Antrim, United Kingdom) according to the manufacturer’s instructions. The reactivity of the antisera was confirmed by use of antigen-positive and negative cells selected from an antibody identification panel (DiaPanel^®^, Lot 45241.88.1, Bio-Rad^®^, Cressier FR, Switzerland). Total and HDL cholesterol measurements were also made using the AU480 chemistry analyser (Beckman-Coulter, Brea, California, United States). Non-HDL cholesterol was calculated as the difference between the two measured values. We also revisited a previous dataset of 261 normal individuals as a control group to examine the same relationships in healthy, HIV-negative individuals^18^

### Statistical analysis

The results were analysed using IBM SPSS version 24 (IBM Corporation, Armonk, New York, United States) statistical software. Statistical analyses included comparison of means according to carriage of the Lutheran antigens, as well as the correlation of data to explore relationships between variables. Results were considered significant only if *p* < 0.05.

## Results

The mean total cholesterol did not differ between controls and HIV-positive patients (mean ± s.d. = 4.02 ± 0.85 vs 3.96 ± 1 mmol/L, *p* = 0.571) (Table 1). Likewise, the mean non-HDL values did not differ between the groups (2.68 ± 0.85 vs 2.88, ± 0.588 mmol/L, *p* = 0.089).

**TABLE 1:** Comparison of total and non-high-density lipoprotein cholesterol in HIV-positive patients and HIV-negative controls, Julia Molefe Clinic, Gaborone, Botswana, December 2016 – February 2017.

Group	*n*	TC (mmol/L)	Non-HDL (mmol/L)
Control	261	4.02 ± 0.85	2.68 ± 0.86
HIV-positive	100	3.96 ± 1	2.88 ± 0.76
*p*-value	-	0.571	0.089

Note: Results are presented as mean ± s.d. (standard deviation).

HDL, high-density lipoprotein; TC, total cholesterol.

Among HIV-positive patients, total cholesterol correlated weakly and *negatively* with monocytes but not with neutrophils (*r* = −0.208, *p* = 0.038 vs *r* = −0.090, *p* = 0.372). However, with respect to the atherogenic non-HDL cholesterol, a stronger depletive effect was observed with monocytes (*r* = −0.401, *p* = 0.006), but not neutrophils (*r* = −0.226, *p* = 0.136). Among HIV-negative controls, there was no correlation between monocytes and total cholesterol or non-HDL cholesterol. However, a very weak *positive* relationship was observed between total cholesterol and neutrophils (*r* = 0.142, *p* = 0.023).

**FIGURE 1:**
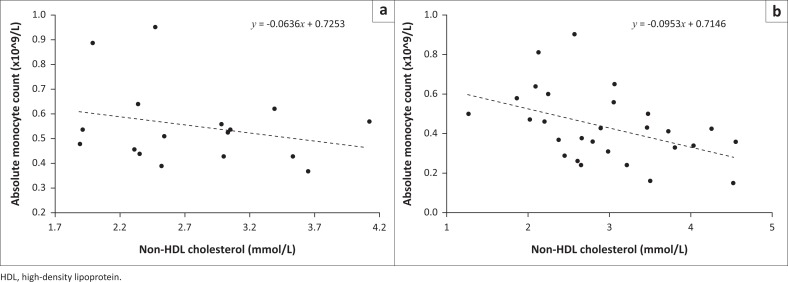
Monocyte depletion in HIV infection was inversely associated with increased non-HDL cholesterol, Julia Molefe Clinic. This relationship was weaker in Lu^b^ antigen-negative patients (a) but enhanced in Lu^b^-positives (b). (a: *r* = −0.259, *p* = 0.315 versus b: *r* = −0.442, *p* = 0.018). Julia Molefe Clinic, Gaborone, Botswana, December 2016 — February 2017.

**FIGURE 2:**
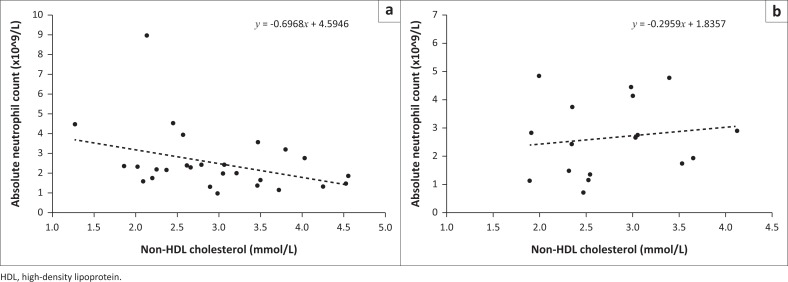
In Lu^b^-positive patients (a), non- high-density lipoprotein cholesterol was associated with neutrophil depletion. This effect was totally absent in Lu^b^-negative patients (b). (a: *r* = −0.369, *p* = 0.053, versus b: *r* = 0.143, *p* = 0.584). Julia Molefe Clinic, Gaborone, Botswana, December 2016 - February 2017.

When patients were segregated according to their Lu^b^ phenotypes, the correlation between non-HDL cholesterol with phagocytes improved for both monocytes (*r* = −0.442, *p* = 0.018; Figure 1) and neutrophils (*r* = −0.369, *p* = 0.053; Figure 2). In contrast, this relationship was absent for both phagocytes in Lu^b^-negative patients (*r* = −0.259, *p* = 0.315 for monocytes vs. *r* = 0.143, *p* = 0.584 for neutrophils). Overall, Lu^b^ expression was associated with lower absolute monocyte counts (0.45 ± 0.18 vs 0.54 ± 0.22 × 10^9^/L, *p* = 0.023).

## Discussion

We studied the relationship between cholesterol and phagocytes in HIV-positive and HIV-negative individuals in an attempt to determine the influence, if any, of the Lu/BCAM on phagocyte counts. We hypothesised that carriage of the Lu^b^ antigen, an adhesion molecule, would enhance margination of leukocytes leading to a peripheral deficiency of the affected leukocytes. Indeed, we observed a significantly lower absolute monocyte count in Lu^b^-positive individuals compared to Lu^b^-negative individuals.

We also observed a negative correlation between cholesterol and phagocytes (neutrophils and monocytes), which corroborates the findings by other investigators that membrane cholesterol, which is in equilibrium with plasma cholesterol,^19^ increases leukocyte adhesion and extravasation.^1,2^ Moreover, phagocyte counts were negatively correlated with non-HDL cholesterol, which is known to promote adhesion and atherogenesis.^1,12^ The monocyte depletive effect of cholesterol was accentuated in Lu^b^-positive individuals but totally absent in the Lu^b^-negative individuals. While the difference in absolute monocyte counts may be considered to be small, the difference translates to 90 million cells per litre. The resulting statistical significance underlines a consistent finding of monocyte depletion in Lu^b^-positive individuals, and may explain the insidious development characteristic of atherosclerosis.

We conclude that higher levels of cholesterol are associated with lower peripheral monocyte and neutrophil counts and that this depletion is accentuated in individuals expressing the Lu^b^ antigen. This depletion is an effect of HIV infection, since it was not observed in the HIV-negative population and probably involves the particular role of the HIV-Tat protein’s enhancement of vascular adhesion and trans-endothelial migration of monocytes reported by other investigators.^20^ HIV-infected monocytes are known to migrate more efficiently across vessel walls, while maintaining their infectivity.^8^ We conclude that the Lu^b^ blood group contributes to the depletion of circulating phagocytes, possibly by enhancing their margination and extravasation.^2^

Working from the established fact that cholesterol, especially non-HDL cholesterol, promotes the adhesion of phagocytes,^1,2^ with the possible reduction of peripheral counts, we note that in HIV infection, even normal cholesterol levels are associated with diminishing circulating phagocytic cells. This could be due to the chronic inflammatory status occasioned by HIV infection. It appears, therefore, that HIV-positive individuals are at higher risk of developing cholesterol-related monocytopenia and neutropenia, which, in the presence of increased non-HDL cholesterol, points to the peculiar vulnerability of HIV-positive patients to cardiovascular diseases. Although these observations were generally expected considering that cholesterol promotes tethering of phagocytes,^2,8^ it was interesting that only in HIV infection was the phagocyte-depleting effect of cholesterol significant.

When cholesterol components were examined, non-HDL cholesterol negatively affected phagocyte counts consistently, especially the monocyte counts, which corroborates previous reports by others^11^ linking non-HDL cholesterol to atherosclerosis in HIV infection.

### Limitations

A limitation of our study was that we did not directly measure phagocyte migration but rather inferred it from diminished peripheral cell counts. Future studies need to focus on direct migration measurements of phenotyped phagocytes. Moreover, the ages of the patients included in the study were not available, making it impossible to include the effect of age in the cholesterol analysis. This notwithstanding, the results provide critical information for HIV-associated atherosclerosis.

### Conclusion

Our findings suggest that the risk of cardiovascular disease in HIV-1-positive patients may in part be due to an interplay between cholesterol, phagocytes and Lu^b^-mediated adhesion, all of which tend to recruit phagocytes out of circulation. While a genetic predisposition for atherogenesis has always been alluded to, our results suggest that the Lu^b^ adhesion molecule may be a potentially important genetic link. The results also suggest a need to manage cholesterol in HIV-positive patients to achieve lower levels than the general public, especially for those who are Lu^b^-positive. Moreover, the vulnerability occasioned by Lu^b^-positive status may provide an opportunity for personalised care in Lu^b^-positive, HIV-positive individuals.
